# From Perception to Action: Air Pollution Awareness and Behavioral Adjustments in Pregnant Women in Serbia

**DOI:** 10.3390/healthcare13121475

**Published:** 2025-06-19

**Authors:** Ana Susa, Milica Zekovic, Dragana Davidovic, Katarina Paunovic, Vera Kujundzic, Sladjana Mihajlovic, Ljiljana Bogdanovic

**Affiliations:** 1 Faculty of Medicine, University of Belgrade, 11000 Belgrade, Serbia; 2 Institute of Hygiene and Medical Ecology, 11000 Belgrade, Serbia; 3 Centre of Research Excellence in Nutrition and Metabolism, Institute for Medical Research, National Institute of Republic of Serbia, University of Belgrade, 11000 Belgrade, Serbia; 4 University Hospital “Dr Dragisa Misovic-Dedinje”, 11000 Belgrade, Serbia; 5 Institute of Pathology, 11000 Belgrade, Serbia

**Keywords:** air pollution, perception, behavioral response, pregnant women, patient-centered care, quality of life

## Abstract

In regions with sustained air pollution, the adoption of protective health behaviors is critical, particularly among pregnant women—a population marked by physiological vulnerability and heightened receptivity to preventive guidance. Understanding and supporting patient-driven behavioral change requires attention to individual perception and awareness, which are shaped by socio-economic and spatial factors, as well as access to credible information. **Objectives**: This study investigates how pregnant women in Serbia perceive air quality, identifies determinants that influence these perceptions, and evaluates the extent and nature of behavioral adaptations undertaken to mitigate exposure-related risks. **Methods**: A cross-sectional survey was conducted among 279 pregnant women using a structured, researcher-administered questionnaire. Collected data included demographic and psychosocial variables, air quality perceptions, self-reported health effects, and behavioral responses. Residential proximity to land-use attributes was assessed using GIS-based spatial analysis. **Results**: Most participants perceived air quality as poor (68.8%), primarily informed by unofficial sources such as mobile applications and social media. Living close to continuous urban fabric (OR = 0.180, 95% CI: 0.059–0.558, *p* = 0.003) and water (OR = 0.306, 95% CI: 0.127–0.738, *p* = 0.008) was associated with poorer perceptions, while proximity to forests (OR = 2.938, 95% CI: 1.323–6.525, *p* = 0.008) correlated with more favorable assessments. Despite prevalent concern, around half of respondents (50.2%) reported no behavioral modifications. Importantly, none had received guidance from healthcare professionals on the topic. **Conclusions**: These findings highlight critical gaps in environmental health literacy and provider engagement. Integrating tailored communication and behavioral support in existing prenatal counseling could advance health-related quality of life in this vulnerable population.

## 1. Introduction

With the escalating global focus on climate change, air pollution has emerged as a critical public health issue, gathering substantial attention from the scientific and medical communities. Exposure to ambient air pollutants is well established as a contributing factor to cardiovascular and respiratory pathologies [1,2,3], with adverse effects observed even at relatively low concentrations over prolonged periods [4]. Beyond these traditional associations, emerging evidence has elucidated the significant role of air pollution in a broader spectrum of health conditions, encompassing diabetes mellitus [5], neurological and neurodevelopmental disorders [6,7], different types of cancer [8,9], as well as in adverse pregnancy and birth outcomes [10,11].

Among other populations, pregnant women are considered to be particularly susceptible to adverse health outcomes resulting from exposure to many environmental factors, including air pollution. This increased vulnerability is attributed to profound physiological adaptations during pregnancy, encompassing significant changes in the respiratory, cardiovascular, and endocrine systems [12]. Recent data indicate that exposure to air pollutants significantly increases the risk of gestational diabetes, gestational hypertension, and preeclampsia [13,14,15]. Furthermore, exposure to pollutants during critical intrauterine development has been linked to adverse health outcomes in the offspring, including low birth weight, prematurity, congenital anomalies, and increased mortality [16,17]. However, of important note is that these studies assess pollution effects based on estimated exposure levels, without accounting for whether individuals adopt protective behaviors, thus potentially underestimating the actual effects.

Beyond these physical health effects, exposure to air pollution may also impair quality of life not only through direct health impacts but also through psychological mechanisms. First, the somatic illnesses, such as gestational diabetes mellitus or gestational hypertension, resulting from pollution exposure may diminish the quality of life [18,19]. Second, exposure to poor air quality in pregnant women may lead to psychological distress, including feelings of annoyance [20], which may further reduce quality of life and overall well-being.

The severity and complexity of these detrimental outcomes have urged policymakers worldwide to prioritize this pressing public health issue, employing both population-level interventions and targeted individual strategies to mitigate its impact. In 2024, the World Health Organization (WHO) published a report that summarized personal-level actions that the general public, as well as vulnerable groups, should implement in order to reduce the negative health effects of air pollution [21]. However, the successful implementation of these protective measures is heavily influenced by social acceptability [22], a factor determined by individual perception and awareness [23]. Socio-demographic factors, including sex, age, education, and income level, alongside spatial determinants such as proximity to green spaces and traffic density, are known to shape perceptions of air quality [24,25,26]. These perceptions, in turn, directly inform the social acceptability and, therefore, adoption of strategies to reduce personal exposure to air pollutants, underscoring the interplay between awareness, perception, and action. Furthermore, Song et al. [25] demonstrated a significant discrepancy between perceived and estimated exposure levels, highlighting the importance of providing clear information to guide public perceptions and, therefore, influence protective behavior strategies.

Several psychological models, such as the Health Belief Model (HBM) and Ajzen’s Theory of Planned Behavior (TPB), focus on understanding these complex interactions and are commonly used in health promotion programs [27,28], as well as in interpreting the multifaceted daily experiences of vulnerable populations [29]. The HBM suggests that individuals are more likely to engage in protective behaviors when they perceive a risk. Similarly, the TPB proposes that attitudes, social norms, and perceived behavioral control shape behavioral intentions and, therefore, determine the extent of protective actions taken to mitigate health risks.

In recent years, Serbia has faced alarming levels of air pollutants, consistently ranking among the most polluted countries in Europe. Based on the report by the European Environment Agency (EEA), the 2022 annual concentrations of particulate matter with aerodynamic diameter less than 10 μm (PM_10_) and less than 2.5 μm (PM_2.5_), ozone (O_3_), and nitrogen dioxide (NO_2_) exceeded the WHO annual limit values at all monitoring stations included in the report [30]. Nevertheless, compliance with the less stringent European Union (EU) and national standards presents an improved scenario. A more in-depth analysis, taking into account all monitoring stations throughout Serbia, has been reported by the Serbian Environmental Protection Agency (SEPA). In the Annual Report of Air Quality in Serbia for 2023, PM_10_ and PM_2.5_ are the most commonly registered air pollutants, exceeding limit values in several monitoring stations, almost all within the urban areas [31]. The capital city of Belgrade faces some of Serbia’s most severe air pollution, creating major health risks for residents, especially in some of its municipalities. While other towns, such as Valjevo, Novi Pazar, and Uzice, may have higher pollutant levels due to coal burning or topography characteristics that cause the accumulation of air pollutants (e.g., situated in valleys), Belgrade holds around a quarter of the entire Serbian population, making the public health implications very alarming [31].

The severe exposure highlights the critical need to address the perceptions and awareness of the population, particularly of vulnerable groups. Pregnant women are generally more inclined to adopt healthy behaviors, a tendency often facilitated by the guidance of healthcare professionals [32], underscoring the importance of delivering precise and accessible information. Understanding how pregnant women perceive air pollution, alongside the societal and spatial factors that shape these perceptions, is essential for designing targeted interventions. Such efforts are pivotal for promoting protective strategies, mitigating exposure, and reducing the health burden of air pollution in this high-risk group. To the best of our knowledge, there are no studies conducted in Serbia or the Balkans that, simultaneously or individually, focus on perception of air quality, adoption of protective behavior, and spatial influences in pregnant women.

This study aims to explore overall perceptions of air quality in pregnant women in Serbia, with a particular focus on identifying the key factors influencing these perceptions, including informational and spatial aspects. Additionally, the study seeks to evaluate which symptoms participants associate with air pollution exposure, as well as analyze which behavioral adjustments they implement to mitigate these effects. Therefore, our goal is to explore the entire route, from societal and spatial factors, through perceptions of air quality and perceived health effects, to the adoption of protective measures, while highlighting the critical points that need to be addressed.

## 2. Materials and Methods

### 2.1. Study Design

This cross-sectional study explores perceptions of air pollution among pregnant women in Serbia. Participants were recruited from the Hospital for Gynecology and Obstetrics, Clinical Center “Dr Dragiša Mišović-Dedinje”, during the period from September 2023 to May 2024, as part of a broader case-control study investigating air pollution as a potential risk factor for gestational diabetes mellitus. For the purposes of this analysis, both cases and controls were treated as a unified population, transcending the initial case-control classification. This approach ensures inclusivity while enhancing the generalizability of findings to the broader population of pregnant women represented within the study.

### 2.2. Study Participants

The sampling method for this study was convenience-based, recruiting participants from a single healthcare center. Eligible participants were pregnant women aged 18 years or older with a confirmed singleton pregnancy, residing in Serbia for at least one year prior to recruitment, and proficient in the Serbian language. Women with chronic medical conditions, such as severe and poorly managed respiratory diseases or psychiatric disorders, as well as multiple pregnancies, were not eligible for the study, as it could influence their perceptions of air pollution. Furthermore, participants with incomplete or insufficient demographic, clinical, or questionnaire data, and women who frequently relocated within the year preceding recruitment, were excluded. Based on these criteria, we included 279 participants who were recruited at the time of delivery.

### 2.3. Ethical Considerations

This study was conducted in full compliance with ethical standards set forth by the Declaration of Helsinki and applicable national regulations governing biomedical research. Ethical approval was obtained from the Ethical Committee of the Clinical Center “Dr Dragiša Mišović-Dedinje” (reference number: 12890/2-2023, date: 20 June 2023), as well as from the Ethical Committee of the Faculty of Medicine (reference number: 17/X-5, date: 24 October 2023). Participants were comprehensively informed about the study’s objectives, procedures, and their rights, including their right to withdraw from the study at any point without penalty or other repercussions. Prior to enrollment, written informed consent was obtained from each participant. The consent process involved both verbal and written explanations of the study, presented in clear and accessible language to ensure participant comprehension. Any questions or concerns were addressed thoroughly during this process. Participant confidentiality and anonymity were rigorously safeguarded through the use of unique identifiers in the dataset, with all personally identifiable information securely protected and accessible only to authorized research personnel.

### 2.4. Pregnancy-Related Data

Information regarding participants’ age, weight gain during pregnancy, Body Mass Index (BMI) at delivery, number of deliveries (including delivery at recruitment), gestational age at delivery, and type of delivery were obtained from hospital records and corroborated through interviews conducted by trained personnel.

### 2.5. Questionnaire

A structured, researcher-administered survey tool—“Pregnant Women’s Air Quality Perception, Impact, and Adaptation Questionnaire (P-AIR-Q)” (Supplementary Material), specifically developed for this study, was utilized to capture comprehensive information on participants’ demographic profiles, perceptions of air quality, behavioral adaptations, and associated health effects. The instrument was designed through an interdisciplinary collaboration involving environmental specialists, gynecologists, public health professionals, and social scientists, thus ensuring its face validity. Additionally, prior to determining its final form, the questionnaire was pilot-tested on a convenience sample of ten participants. Following the feedback and the integration of suggestions from both the expert panel and the participants, the survey tool ultimately comprised 20 items organized into four distinct sections:

Section 1: Demographic, Lifestyle, Residential, and Socioeconomic Characteristics

The first section gathered detailed demographic and lifestyle data, including age, smoking status in households, residential address, type of residence, residential floor level, and window orientation. Socioeconomic indicators were also collected, encompassing employment status, level of formal education, and perceived family income. The urban–rural typology was determined by participants’ residential addresses using the Organization for Economic Co-operation and Development (OECD) classification [33] and the Statute of Belgrade [34].

Section 2: Perception of Air Quality and Information Engagement

The second section explored participants’ subjective appraisal of air quality and their interaction with various official and unofficial informational resources. Information reported directly by agencies and institutions monitoring and reporting on air quality (Serbian Environmental Protection Agency, the Institute of Public Health of Belgrade, or the Institute of Public Health of Serbia) is considered official, whereas information provided by third-party sources, such as different webpages, applications, and media, is considered unofficial.

Section 3: Behavioral Adjustments and Recommendations

This section examined participants’ behavioral responses and strategies to mitigate the effects of poor air quality. Participants were asked, “Do you change your habits when the air quality is low?” with multiple-choice response options. Multiple responses were allowed to capture the full range of adaptive strategies employed. Next, we inquired whether they were familiar with the recommendations regarding protective measures and, if so, what were the sources of information.

Section 4: Perceived Health Effects and Psycho-Emotional Distress

The fourth section investigated health symptoms that participants attributed to poor air quality. Participants were asked, “Do you have any symptoms that you would attribute to air pollution?” If the response was affirmative, further inquiries addressed the nature of symptoms, whether they exhibited a seasonal pattern, and whether they subsided when indoors. Lastly, psycho-emotional distress was assessed with the question, “Does air pollution cause you any fear or psychological discomfort?”.

To assess the questionnaire’s reliability, we analyzed dichotomous items and dichotomized multiple-response items, calculating a Kuder-Richardson 20 (KR-20) coefficient of 0.794, indicating acceptable to good internal consistency.

### 2.6. Geospatial Assessment of Proximity to Land Use Attributes

The geospatial proximity to land use attributes was assessed using geometric (straight-line) calculations. To achieve this, data from two comprehensive land use datasets were utilized: the Urban Atlas Land Cover/Land Use 2018 and the CORINE Land Cover 2018. For regions with populations exceeding 50,000 inhabitants, four land use attributes were extracted from the Urban Atlas dataset: urban green areas, forests, water bodies, and continuous urban fabric (>80% impervious surface).

For regions with fewer than 50,000 inhabitants, where Urban Atlas coverage is unavailable, the CORINE Land Cover dataset was utilized. Attributes derived from this dataset included urban green areas, forests (further classified into broad-leaved, coniferous, and mixed forest categories), water features (distinguished as water courses and water bodies), and continuous urban fabric (>80% impervious surface).

Following the geocoding of each participant’s residential address, proximity to the identified land use attributes was calculated within a Geographic Information System (GIS) framework, utilizing the Euclidean Distance tool in ESRI ArcGIS Pro (version 3.2.1). Data on the proximity to land use attributes is further classified into quartiles (urban green areas: Q1 ≤ 180.3 m, Q2 = 180.4–335.4 m, Q3 = 335.5–807.8 m, and Q4 ≥ 807.9 m; forest: Q1 ≤ 626.5 m, Q2 = 626.6–1150.0 m, Q3 = 1150.1–1843.9 m, and Q4 ≥ 1844.0 m; water: Q1 ≤ 1141.3 m, Q2 = 1141.4–2607.7 m, Q3 = 2607.8–4245.0 m, and Q4 ≥ 4245.1 m; continuous urban fabric: Q1 ≤ 30.0 m, Q2 = 30.1–194.2 m, Q3 = 194.3–1150, and Q4 ≥ 1150.5 m).

### 2.7. Statistical Analysis

Continuous variables regarding demographic and pregnancy-related characteristics are presented as means (SD), whereas variables regarding proximity to land use attributes are presented as medians (IQR). Categorical variables are presented as frequencies and percentages. A combination of non-parametric and multivariable modeling techniques was used to explore associations between participants’ sociodemographic and residential characteristics, proximity to land use attributes, perception of air quality, and behavioral adjustments. Spearman’s rank-order correlation was used to assess monotonic relationships between ordinal variables and perceived air quality. This method was appropriate, given the ordinal nature of many key variables (e.g., education level, level of formal education, residential proximity to land use attributes) and the absence of a linearity assumption. Additionally, for dichotomous categorical predictors, such as employment status or type of residence, we applied the Mann–Whitney U test to evaluate group differences in the distribution of perception scores. This non-parametric test is suitable for comparing the distribution of ordinal outcomes between two independent groups. In addition, to evaluate the combined influence of multiple sociodemographic and spatial variables, we performed logistic regressions with perception of air quality and adoption of protective behaviors as outcome variables. In these analyses, the variable perception of air quality was dichotomized into poor vs. fair/good due to limited responses in one category. Independent variables in regression models included sociodemographic and residential variables, as well as categories of proximity to land use attributes. Additionally, logistic regression was utilized to test the associations between reported psycho-emotional distress and urban–rural typology, while accounting for sociodemographic characteristics. A *p* value of less than 0.05 was considered significant. This analysis was conducted using R 4.3.0 software (R Foundation for Statistical Computing, Vienna, Austria).

## 3. Results

### 3.1. Demographic and Clinical Data

A total of 279 pregnant women were enrolled in this study. The mean age of participants was 31.8 ± 4.9 years. Detailed demographic and clinical characteristics related to pregnancy are summarized in Table 1.

### 3.2. Lifestyle, Residential, and Socioeconomic Characteristics

The majority of participants (96.4%) reported residing within the Belgrade agglomeration. Of the total cohort, 212 women (76.0%) lived in urban settings, while 67 (24.0%) resided in suburban or rural areas. Key self-reported lifestyle, residential, and socioeconomic characteristics are presented in Table 2.

### 3.3. Perception of Air Quality and Information Engagement

**Figure 1:**
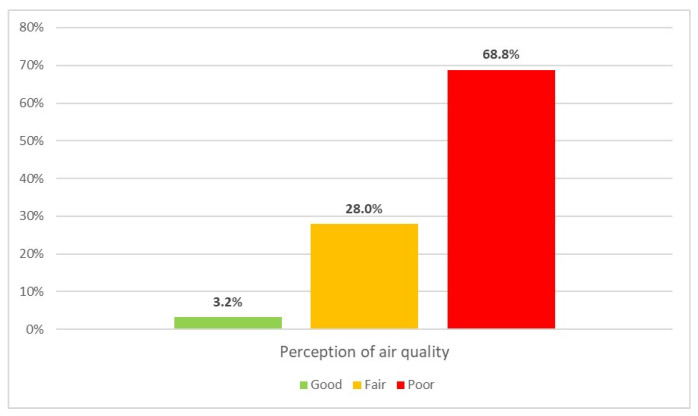
The relative proportions of different perceptions of air quality in participants’ home cities (N = 279).

Figure 1 illustrates participants’ overall perception of air quality within their home cities or neighborhoods. The perception of air quality did not correlate with participants’ age (*p* = 0.075). The Mann–Whitney-U test indicated that urban residents reported significantly poorer air quality compared to suburban residents (U = 5570.5, *p* = 0.001; with a small effect size—*r* = −0.22). Similarly, those living in apartment buildings reported worse air quality perception than those living in houses (U = 11,248.00, *p* < 0.001; with a small- effect size—*r* = 0.20). Spearman’s correlation indicated that those who have windows overlooking the street (*p* = 0.013) have a markedly poorer perception of air quality, although weakly associated (ρ = −0.148). No statistically significant correlation was found between the perception of air quality and residential floor level (*p* = 0.575).

Lastly, the perception of air quality was associated with all self-reported socioeconomic indicators. Poor air quality is perceived more often by participants reporting a higher level of formal education (*p* = 0.010; weak association—ρ = 0.154). Similarly, perceptions of air quality varied significantly depending on employment status (U = 6825.5, *p* = 0.029, with a small effect size—*r* = 0.16) and perceived family income (U = 7655.5, *p* < 0.001; with a small to medium effect size—*r* = −0.21), indicating that those who were employed and report higher family income more often perceive air quality as poor.

Regression analysis of the associations between sociodemographic and residential characteristics and the perception of air quality is reported in Table 3. Among the tested variables, only perceived family income emerged as significant (*p* = 0.016), with higher income associated with 53% lower odds of good/fair air quality perception (OR = 0.470, 95% CI: 0.255–0.876).

**Figure 2:**
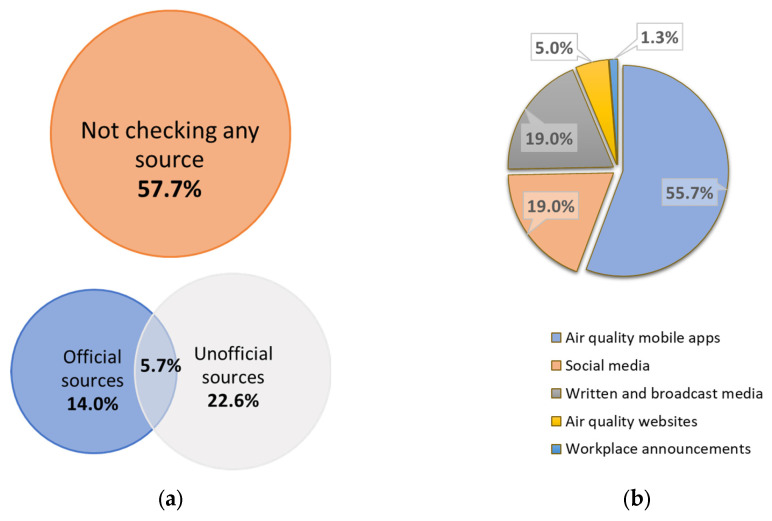
Reported information engagement regarding the air quality of the study population. (**a**) The habits of checking air quality information with the type of sources checked (N = 279). (**b**) The preferred types of unofficial sources (N = 79).

Information engagement was evaluated through participants’ reliance on both official and unofficial information sources regarding air quality. When asked about their use of official channels, only a minority (55 participants; 19.7%) indicated affirmative engagement. A slightly higher proportion of participants (79 individuals; 28.3%) reported consulting unofficial sources for air quality information. Among the unofficial options, mobile applications specifically designed for air quality reporting were the most commonly utilized, with 55.7% of respondents identifying them as their preferred source. These findings, along with a detailed breakdown of the types of unofficial sources accessed, are presented in Figure 2.

### 3.4. Behavioral Adjustments and Recommendations

**Figure 3:**
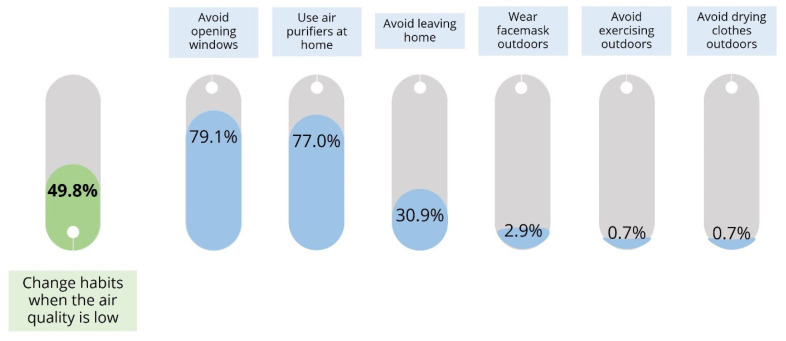
The relative proportion of participants who report behavioral adjustments during periods of poor air quality (pictured in green; N = 279) along with the proportions of each reported adjustment (pictured in blue; N = 139).

When prompted about the behavioral adjustments during periods of poor air quality, about half of the participants reported not undertaking any protective measures (50.2%). Among those who did, the majority focused on actions aimed at improving indoor air quality. The most frequently reported measures included avoiding window openings during periods of poor air quality (79.1%) and the use of air purifiers within the home environment (77.0%). The majority of participants relied on two adjustments concurrently (68.4%), a smaller proportion reported employing one protective strategy (20.1%), while 16 individuals (11.5%) indicated engaging in three distinct protective habits. The distribution of behavioral adjustments during poor air quality episodes, along with the specific types of measures undertaken, is presented in Figure 3. The represented proportions of each adjustment type are relative to the subset of participants who engaged in behavioral modifications.

Women were also asked if they were informed about recommendations regarding protective strategies to alleviate the effects of air pollution as well as what the sources of these recommendations were. Strikingly, a majority of respondents (160 participants; 57.3%) reported being unfamiliar with any form of recommendation. Among those who were aware of protective recommendations, the predominant sources were media outlets and internet searches, referenced by one hundred and six participants (89.1%). A notably smaller proportion identified their workplace as a source of information (thirteen participants; 10.9%), while informal discussions with family and friends were cited by only five individuals (4.2%). None of the participants reported consulting their physician, including, but not limited to, general practitioners, obstetrician-gynecologists, or other specialists involved in maternity care, for advice on mitigating the effects of air pollution, and none reported being a part of any air pollution mitigation intervention intended for pregnant women. Furthermore, only a small number of informed participants (four individuals; 3.4%) exhibited proactive engagement by consulting more than one source.

### 3.5. Perceived Health Effects and Psycho-Emotional Distress

During the interview, approximately one-fifth (58 participants; 20.8%) responded that they experienced health symptoms that could be attributed to air pollution. Women most often reported respiratory symptoms (54 participants; 93.1%), such as cough, shortness of breath, and frequent upper respiratory tract infections. Eye irritations (three participants; 5.2%) and nausea stemming from unpleasant odors (one individual; 1.7%) were rarely reported. Unsurprisingly, these symptoms followed a seasonal pattern, with the most common occurrence during winter (thirty-five participants; 60.4%) and autumn (ten participants; 17.2%) months, and less common in spring (eight individuals; 13.8%) and summer (five individuals; 8.6%). Furthermore, most participants reported subsiding of symptoms when indoors, either completely (thirty-nine participants; 67.2%) or partially (sixteen participants; 27.6%), while only three respondents (5.2%) reported that these symptoms persisted.

Lastly, we raised interest in psycho-emotional distress by inquiring about participants’ feelings of fear, anxiety, or any other psychological discomfort caused by the poor air quality. Even though the majority of respondents perceived air quality as poor, only 37 (13.3%) reported having fear or psychological discomfort regarding the health effects of air pollution. The presence of psycho-emotional distress was not associated with residing in urban and suburban/rural environments, even after accounting for sociodemographic characteristics, such as employment status, level of formal education, and perceived family income (β = −0.165, OR = 0.848, 95% CI: 0.359–2.006, *p* = 0.708).

### 3.6. Geospatial Assessment of Proximity to Land Use Attributes

**Figure 4:**
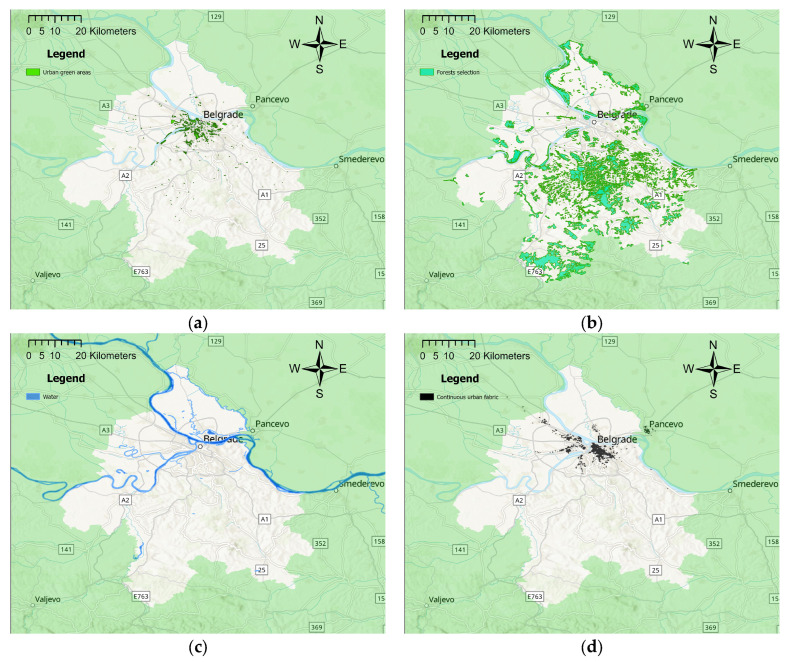
Spatial distribution map of (**a**) urban green areas, (**b**) forests, (**c**) water, and (**d**) continuous urban fabric within the Belgrade agglomeration adapted from the Urban Atlas Land Cover/Land Use 2018 and the CORINE Land Cover 2018 datasets.

Figure 4 presents spatial distributions of four land use attributes within the Belgrade agglomeration, from which straight-line distance was calculated. The overall Euclidean distance between participants’ home addresses and these attributes is presented in Table 4.

The proximity to urban green areas exhibited a statistically significant negative correlation with air quality perception (*p* = 0.004, with a negligible relationship—ρ = −0.170), indicating that individuals living closer to these areas were more likely to report poor air quality. Similar to urban green areas, respondents living closer to water bodies more often perceive air quality as poor (*p* ≤ 0.001, with a weak relationship—ρ = −0.242). Conversely, residing closer to forests is positively associated with perceiving air quality as good (*p* = 0.001, with a weak relationship—ρ = 0.206). Lastly, proximity to continuous urban fabric emerged as the strongest predictor of participants’ perception of air quality among all analyzed land use attributes. Participants living in closer proximity to highly urbanized areas were significantly more inclined to report poor air quality (*p* ≤ 0.001, with a moderate relationship—ρ = −0.328). Table 5 provides a detailed overview of the participants’ perceptions of air quality in relation to their proximity to analyzed land use attributes.

Regression analysis of the combined associations between perception of air quality and straight-line proximity to land use attributes is summarized in Table 6. For forests, greater distance significantly worsens air quality perception, with residents farthest away (Q4) showing nearly three times higher odds of poor perception compared to those living closest (OR = 2.938, 95% CI: 1.323–6.525, *p* = 0.008). On the other hand, water shows the opposite effect, where closer proximity is associated with poorer perception (Q4 OR = 0.306, 95% CI: 0.127–0.738, *p* = 0.008), indicating 69% lower odds of poor perception at farther distances. Similarly, those residing further from the continuous urban fabric (Q4) have significantly better air quality perceptions, with 82% lower odds of poor rating compared to those living closest (OR = 0.180, 95% CI: 0.059–0.558, *p* = 0.003).

### 3.7. Regression Analysis of Behavioral Adjustments

To identify the strongest predictors of behavioral adjustments, we constructed multiple models (Table 7). Model 1 showed that participants who reported air quality as poor are more likely to adopt behavioral change (OR = 1.563, 95% CI: 1.050–2.936, *p* = 0.032). However, this association became insignificant in adjusted models. Model 2 shows that the type of residence was the strongest predictor, with those residing in apartments showing 2.6 times higher odds of changing habits compared to those residing in houses (OR = 2.626, 95% CI: 1.397–4.937, *p* = 0.003). This effect became even stronger in the fully adjusted Model 4 (OR = 3.265, 95% CI: 1.627–6.552, *p* = 0.001), where living in an apartment was associated with 3.3 times higher odds. Proximity to urban green spaces showed inverse effects in Model 3, with residents in Q2 (OR = 0.440, 95% CI: 0.214–0.904, *p* = 0.025) and Q4 (OR = 0.366, 95% CI: 0.153–0.869, *p* = 0.024) being less likely to adopt protective actions compared to the ones that reside closest to urban green areas. However, these effects attenuated after the full adjustment in Model 4. The fully adjusted model also showed marginal trends for suburban residence (OR = 0.379, 95% CI: 0.142–1.010, *p* = 0.052) and lower income (OR = 0.549, 95% CI: 0.301–1.003, *p* = 0.051) being associated with lower odds of habit change.

## 4. Discussion

Understanding how individuals perceive environmental risks and translate them into behavioral responses is essential for shaping effective public health strategies. In the context of air pollution, this relationship becomes particularly significant for vulnerable populations, such as pregnant women, whose exposure can have both immediate and long-term health implications. This study examines the intersection of environmental characteristics, cognitive appraisal, and behavioral adaptation within this high-risk group, offering insights into how spatial factors, informational access, and individual perceptions interact to shape health-related decision-making. The findings underscore the complexity of air quality perception, offering an empirical foundation for reimagining health promotion, environmental risk communication, and interdisciplinary care in ways that meaningfully enhance quality of life.

### 4.1. Perceptions in Light of Current Air Quality in Serbia

One of the most prominent findings of this study was the widespread perception of poor air quality among participants, a reflection of the growing public concern over air pollution in Serbia, where environmental conditions have been undergoing rapid and significant changes in recent years. To date, efforts have been directed toward aligning Serbian regulations with European Union legislation, especially concerning the annual limit values [35]. Furthermore, air quality monitoring infrastructure in Serbia has expanded substantially, particularly in urban centers, increasing from 39 fixed stations in 2010 to 275 stations and sites by 2023 [31,36]. The expanded monitoring network has illuminated the severity of the situation, reinforcing the urgency of addressing this public health concern. Based on the findings, in 2022, the government adopted the “Air quality programme of the Republic of Serbia for the period 2022–2030 with an action plan” intended to focus on reducing air pollution on a policy level. This program detailed measures and their environmental impacts, and stated “education on air quality, training for implementation of best practices and awareness raising” as one of the objectives [37].

Although this study did not provide actual pollution data in relation to perceptions of air quality, the objective presence of pollutants should remain a fundamental driver of air quality perceptions. Apart from that, broader social and structural factors intricately shape how individuals interpret these environmental conditions. A comprehensive study spanning 26 European countries showed that economic inequalities could also play an important role in perceiving air quality as poor [38].

### 4.2. Understanding the Relationship Between Informational Accessibility, Perception, and Behavioral Responses Using Psychological Models

The findings of this study can be contextualized within the frameworks of the Health Belief Model (HBM) and Ajzen’s Theory of Planned Behavior (TPB), two complementary psychological models for understanding health-related behaviors. While the HBM focuses on cognitive constructs such as perceived susceptibility, severity, benefits, barriers, self-efficacy, and cues to action [27], the TPB highlights the role of intention, attitudes toward the behavior, subjective norms, and perceived behavioral control in shaping decision-making [28]. Together, these models offer a comprehensive lens for interpreting pregnant women’s perceptions of air pollution and their behavioral responses.

Our findings reveal significant correlations between proximity to specific land use attributes and perceptions of air quality. Residing near continuous urban fabric and water correlated with poorer perceptions, likely reflecting heightened sensitivity to pollution sources in densely urbanized environments, which geographically correspond to near-water areas (confluence of the Danube and Sava rivers). In contrast, proximity to forests correlated with more favorable perceptions, which may highlight the influence of natural surroundings in mitigating concerns about environmental conditions. Similar results are reported in another study [25], where green space and transportation density positively correlate with perceiving air quality as poor. These patterns align with the HBM, wherein perceived susceptibility and severity influence how individuals assess environmental threats and their potential impact.

The effects of land use attributes on air pollution are thoroughly examined and are, therefore, an important subject in urban planning designs. In particular, green infrastructure has been one of the most discussed topics when it comes to mitigating urban air pollution. Urban greenery can not only reduce levels of air pollutants but also act as a barrier to air pollutant dispersion [39]. Apart from its direct environmental benefits, green infrastructure could improve overall well-being and boost confidence and support in communities [40].

In contexts where urban planning fails to prioritize green infrastructure or other policy-level interventions, vulnerable groups, such as pregnant women, must rely on individual protective strategies to reduce risks. However, the extent of these efforts is often insufficient. As observed in this study, regardless of the widespread perception of poor air quality, over half of participants (50.2%) reported not undertaking any protective action. This might point to the possible gaps in perceived severity or confidence in the efficacy of available interventions. However, it appears that perceiving air quality as poor would not motivate pregnant women to adopt protective habits. Instead, mere living in apartment buildings is a stronger predictor of willingness to implement protective actions. The reason for this could lie in differences in overall well-being and behavioral practices between apartment and house residents. This is supported Berglund et al., whose study demonstrated that those residing in apartments (condominiums) are more likely to report poor health or poor psychological well-being when compared to private house residents. Furthermore, when it comes to behavioral changes, apartment residents were more likely to refrain from going out due to safety concerns compared to house residents [41]. The lower overall well-being in those residing in apartments may reflect higher perceived susceptibility of the HBM, resulting in increased engagement in protective practices. Simultaneously, TPB’s subjective norms might be amplified in an apartment setting where dense living increases social observability. Developing targeted intervention strategies while accounting for these characteristics could increase both awareness and implementation of protective changes. However, these interventions should always incorporate actual pollution data when determining the most vulnerable subgroups.

Despite the expansion of air quality monitoring networks and increased public awareness campaigns in Serbia, our findings highlight a notable gap in engagement with official information channels, with only 19.7% of participants consulting institutional reports. This low engagement may stem from several factors, including limited trust in official sources or a lack of effective dissemination strategies tailored to the public’s needs. The preference for unofficial channels, such as mobile applications, underscores the growing demand for user-friendly, real-time, and easily accessible information. These results are similar to the findings of a study from China, where participants reported being more likely to check air quality on TV, newspapers, or mobile phones compared to official channels of the environmental department [42]. The underutilization of official resources poses challenges for public health efforts, as these channels often provide the most accurate and scientifically validated data. To address this, it is imperative to bridge the gap between institutions and the public through tailored health communication strategies. This could include increasing the visibility and credibility of institutional reports, employing clear and relatable messaging, and leveraging popular platforms such as mobile apps or social media to disseminate official data. Furthermore, fostering trust through transparent reporting, community involvement, and collaboration with healthcare professionals could enhance public confidence and encourage broader engagement with official resources. These efforts must also account for self-efficacy, another key construct of the HBM. Empowering individuals—especially vulnerable groups such as pregnant women—with the confidence and skills to access, interpret, and act on official air quality information is critical for fostering behavior change. Strengthening the integration of institutional resources into public health strategies can help align perceptions and behaviors with evidence-based recommendations.

The results from our study raise concerns regarding communication between healthcare professionals and pregnant women about air pollution, as none of the participants reported discussing the issue with their doctor. Similarly, a study conducted in Poland reported that only 3% of doctors inform their patients during episodes of poor air quality [43]. According to another study, none of the obstetricians or gynecologists reported speaking to pregnant women about limiting their exposure to air pollution [44]. The absence of healthcare providers as key sources of information underscores a critical missed opportunity for leveraging cues to action. TPB’s emphasis on subjective norms is particularly relevant here; the lack of normative support from trusted authorities, such as physicians, may impede behavioral intentions to adopt protective strategies. Enhancing healthcare providers’ role in disseminating actionable guidance could not only strengthen self-efficacy, as outlined in the HBM, but also foster positive social reinforcement, a core principle of TPB. Through continuing medical education of healthcare personnel on air pollution protection actions, and the existing infrastructure, such as primary care-based “Schools for Pregnant Women”, these interventions could become a part of routine prenatal counseling.

The limited psycho-emotional distress reported by participants, despite their predominantly negative perception of air quality, underscores a notable disconnect between cognitive awareness and affective responses. This divergence may reflect desensitization to chronic exposure or cultural normalization of poor air quality. Although we may hypothesize that this desensitization could come as a result of a resilience to difficulties, which is often observed in the Balkan region, it is more likely a result of high exposure to negative air pollution data and the subsequent emotional fatigue [45]. Within the HBM framework, this implies limited translation of perceived susceptibility and severity into affective engagement. While participants may cognitively acknowledge air pollution as a health threat, the absence of strong emotional responses suggests that the perceived immediacy or personal relevance of its impact is not sufficient to elicit psycho-emotional distress or drive protective behaviors. Similarly, the TBP underscores the role of attitudes and subjective norms in shaping intentions—factors that may be underdeveloped in this population due to limited social reinforcement or normative pressure to act. To address these gaps, integrating socio-environmental factors—such as cultural attitudes, economic constraints, and community-level influences—into intervention strategies is critical. By leveraging the HBM’s emphasis on perceived benefits and barriers, alongside the TPB’s focus on attitudes and normative influences, tailored interventions can foster a deeper emotional connection to the issue while encouraging more consistent behavioral responses. This dual-theory approach offers a more holistic pathway for designing impactful public health interventions that resonate cognitively and emotionally with vulnerable populations. However important psycho-emotional distress may be for the implementation of protective behaviors, it still brings concern regarding the mental well-being of individuals. Recent studies have shown that air pollution, independent of perceptions, may be associated with the increased prevalence of stress, anxiety, and depression, therefore significantly affecting the quality of life [46,47,48]. This highlights the need for future research on establishing the connection and direction between air pollution perception, exposure, and mental well-being.

### 4.3. Limitations and Future Research Directions

While this study provides important insights into pregnant women’s perceptions of air pollution and associated behaviors, certain limitations warrant acknowledgment to contextualize the findings and inform future research directions. The study’s cross-sectional design, while valuable for capturing a snapshot of attitudes and actions at a specific time, inherently limits the ability to infer causal relationships. Furthermore, as the study was conducted at a single center and predominantly included women from the Belgrade metropolitan area, the findings may not be fully generalizable to pregnant women in other parts of Serbia, especially those living in rural or less urbanized regions. However, focusing on participants from the nation’s capital, where air pollution is especially severe [31], allowed for an in-depth exploration of challenges specific to urbanized and densely populated environments. Additionally, to our knowledge, this is the first study to investigate this topic in Serbia. While this provides a novel contribution to the literature, it also underscores the need for further research to validate and expand upon these findings in different geographical and socioeconomic contexts. Future studies should aim to examine variations in perception and behavior across diverse populations, including rural and lower-income communities. Another limitation lies in the reliance on self-reported data, which is susceptible to biases such as recall and social desirability bias [49]. Although the use of a rigorously designed, pilot-tested questionnaire ensured clarity and consistency, it remains possible that some participants over- or under-reported their behaviors or perceptions. To address this, future research could incorporate objective measures such as personal exposure monitoring devices or real-time tracking of behavioral adjustments to validate and complement self-reported data. The geospatial analysis in this study, though providing a significant understanding of the relationship between residential proximity and land use attributes, is subject to inherent limitations. The reliance on Euclidean distances, which calculate straight-line proximity, may not accurately reflect participants’ actual environmental exposures or accessibility, as it does not account for road networks, physical barriers, or variations in air pollution dispersion [50]. Employing advanced geospatial techniques, such as network-based analyses [51] or integrating localized air quality monitoring data [52,53], could enhance the precision and applicability of future studies. Finally, the temporal scope of the study, conducted from September to May, introduces a potential seasonal bias, as air pollution levels and public awareness may vary across the year. Nevertheless, this period encompasses months when air pollution in Serbia is typically at its peak, offering critical insights into participants’ experiences during high-exposure seasons.

## 5. Conclusions

The ever-growing number of studies regarding air pollution, coupled with an increase in air quality monitoring and media coverage, has led to a growth in public awareness, especially in populations susceptible to adverse health outcomes. Pregnant women, often recognized for their proactive engagement in healthy behaviors, present a unique demographic whose protective practices are shaped by a myriad of factors—most notably, their perceptions of environmental risks. This study underscores the intricate interplay between environmental characteristics, informational accessibility, and behavioral responses in shaping health-related decisions in this population. While negative perceptions of air quality were prevalent, a notable gap emerged in the adoption of protective strategies, highlighting the pivotal role of perception in driving behavioral change. Enhancing targeted public health interventions through continuing medical education of healthcare professionals and the existing infrastructure of prenatal care, such as primary care-based “School for Pregnant Women”, could bridge these gaps and foster protective practices, ultimately leading to disease prevention, improved quality of life, and overall well-being. By addressing the social, cognitive, and emotional dimensions, such interventions should be tailored to resonate with the unique needs of pregnant women, fostering not only greater awareness but also a stronger sense of self-efficacy.

In light of the evolving paradigm of patient-centered care, these findings emphasize the imperative to reframe environmental health as an integral component of personalized, anticipatory, and equity-informed healthcare. Addressing air pollution exposure among pregnant women in dense urban settings, such as Belgrade, cannot rely solely on regulatory or population-level approaches; rather, it requires multidimensional strategies that integrate clinical counseling, behavioral support, and context-specific communication. By illuminating how spatial, informational, and psychosocial determinants converge to shape perceived vulnerability and adaptive behavior, this study offers insights that may contribute to the development of strategies to enhance health-related quality of life. While these findings suggest the potential value of interdisciplinary, evidence-informed interventions tailored to the needs of at-risk groups in similar contexts, further research in diverse populations that incorporate actual pollution data is needed for their development.

## Figures and Tables

**Table 1:** Demographic and pregnancy-related characteristics of the study population (N = 279).

Characteristics	Mean/N	SD/%
Age (years) (mean/SD)	31.8	4.9
Weight gain during pregnancy (kg) (mean/SD)	14.3	7.4
Body Mass Index at delivery (kg/m^2^) (mean/SD)	28.4	4.3
Total number of deliveries (mean/SD)	1.5	0.7
Gestational weeks at the time of delivery (mean/SD)	38.9	1.3
Type of delivery (N/%)		
Vaginal delivery	190	68.1
Cesarean section	89	31.9

**Table 2:** Self-reported lifestyle, residential, and socioeconomic characteristics of the study population (N = 279).

Characteristics	Number of Participants (N)	Percentage of Participants (%)
Lifestyle		
Smoking status		
No	200	71.7
Yes	29	10.4
Former smokers	50	17.9
Smoking in households		
No	143	51.2
Yes	78	28.0
Yes, but not in the vicinity/room	58	20.8
Residence		
Type of residence		
House	113	40.5
Apartment building	166	59.5
Residential floor level		
Ground to 2nd	195	69.9
3rd to 5th	73	26.2
6th and higher	11	3.9
Window orientation		
Overlooking the street	95	34.1
Overlooking the yard	94	33.7
Both	90	32.2
Socioeconomic indicators		
Employment status		
Employed	227	81.4
Unemployed	52	18.6
Level of formal education		
Elementary/middle/high school	86	30.9
Bachelor’s degree	150	53.8
Postgraduate education	43	15.4
Perceived family income		
Good	133	47.7
Fair/poor	146	52.3

**Table 3:** Regression analysis of the associations between air quality perceptions and sociodemographic and residential characteristics (N = 279).

	β *	*p* Value *	OR *	95% CI *
Residential characteristics				
Urban–rural typology	−0.429	0.210	0.651	0.333–1.274
Type of residence	0.398	0.239	1.489	0.768–2.886
Window orientation (ref: overlooking the street)				
Overlooking the yard	−0.111	0.750	0.895	0.453–1.768
Both	0.230	0.348	0.711	0.348–1.445
Sociodemographic characteristics				
Employment status	0.071	0.800	1.074	0.518–2.226
Level of formal education (ref: elementary/middle/high school)				
Bachelor’s degree	0.248	0.469	1.281	0.655–2.506
Postgraduate education	−0.122	0.800	0.885	0.344–2.275
Perceived family income	−0.754	0.016	0.470	0.255–0.876

* Adjusted for age.

**Table 4:** The average (median and IQR) straight-line distance between participants’ residential addresses and land use attributes (N = 279).

	Euclidian Distance from Participants’ Residential Addresses (m)	
Land Use Attribute	Median	IQR
Urban green area	335.4	628.5
Forests	1150.0	1217.4
Water	2607.7	3103.7
Continuous Urban Fabric	194.2	1120.4

**Table 5:** The distribution of air quality perception ratings across categories of distance to land use attributes (N = 279).

	Perception of Air Quality N (%)			
Land use attribute	Good	Fair	Poor	Spearman’s correlation
Urban green areas				
Q1 ≤ 180.3 m	1 (1.6)	18 (28.1)	45 (70.3)	ρ = −0.170 **
Q2 = 180.4–335.3 m	0 (0.0)	14 (18.4)	62 (81.6)	
Q3 = 335.5–807.8 m	3 (4.3)	18 (26.1)	48 (69.6)	
Q4 ≥ 807.9 m	5 (7.1)	28 (40.0)	37 (52.9)	
Forests				
Q1 ≤ 626.5 m	5 (7.0)	32 (45.1)	34 (47.9)	ρ = 0.206 **
Q2 = 626.6–1150.0 m	3 (4.4)	13 (19.1)	52 (76.5)	
Q3 = 1150.1–1843.9 m	1 (1.4)	15 (21.4)	54 (77.2)	
Q4 ≥ 1844.0 m	0 (0.0)	18 (25.7)	52 (74.3)	
Water				
Q1 ≤ 1141.3 m	0 (0.0)	12 (17.4)	57 (82.6)	ρ = −0.242 ***
Q2 = 1141.4–2607.7 m	1 (1.4)	19 (26.8)	51 (71.8)	
Q3 = 2607.8–4245.0 m	2 (2.9)	19 (27.5)	48 (69.6)	
Q4 ≥ 4245.1 m	6 (8.6)	28 (40.4)	36 (51.4)	
Continuous urban fabric				
Q1 ≤ 30.0 m,	0 (0.0)	8 (11.4)	62 (88.6)	ρ = −0.328 ***
Q2 = 30.1–194.2 m	0 (0.0)	20 (29.0)	49 (71.0)	
Q3 = 194.3–1150 m	1 (1.4)	20 (28.6)	49 (70.0)	
Q4 ≥ 1150.5 m	8 (11.4)	30 (42.9)	32 (45.7)	

** *p* ≤ 0.01; *** *p* ≤ 0.001.

**Table 6:** Logistic regression analysis of the associations between the perception of air quality and Euclidean distance of land use attributes (N = 279).

Land Use Attributes	β *	*p* Value *	OR *	95% CI *
Urban green areas (ref: Q1)				
Q2	0.557	0.210	1.747	0.730–4.179
Q3	0.416	0.329	1.516	0.657–3.500
Q4	0.301	0.537	1.351	0.520–3.512
Forests (ref: Q1)				
Q2	1.054	0.009	2.868	1.294–6.355
Q3	0.950	0.021	2.585	1.152–5.800
Q4	1.078	0.008	2.938	1.323–6.525
Water (ref: Q1)				
Q2	−0.702	0.112	0.495	0.208–1.117
Q3	−0.830	0.064	0.436	0.181–1.049
Q4	−1.183	0.008	0.306	0.127–0.738
Continuous urban fabric (ref: Q1)				
Q2	−1.023	0.034	0.360	0.139–0.928
Q3	−0.896	0.069	0.408	0.155–1.072
Q4	−1.714	0.003	0.180	0.059–0.558

* Adjusted for age.

**Table 7:** Logistic regression analysis of factors influencing the adoption of protective behaviors (N = 279).

Characteristics	Model 1 (Perception Unadjusted)		Model 2 (Perception + Sociodemographic + Residential Characteristics)		Model 3 (Perception + Proximity to Land Use Attributes)		Model 4 (Fully Combined)	
	OR (95% CI)	*p* Value	OR (95% CI)	*p* Value	OR (95% CI)	*p* Value	OR (95% CI)	*p* Value
Air quality perception	1.563 (1.050–2.936)	0.032	1.333 (0.759–2.343)	0.317	1.626 (0.906–2.920)	0.103	1.500 (0.801–2.809)	0.205
Urban–rural typology	—	—	0.694 (0.352–1.368)	0.291	—	—	0.379 (0.142–1.010)	0.052
Type of residence	—	—	2.626 (1.397–4.937)	0.003	—	—	3.265 (1.627–6.552)	0.001
Window orientation (ref: overlooking the street)								
Overlooking the yard	—	—	1.104 (0.600–2.032)	0.751	—	—	1.091 (0.576–2.069)	0.567
Both	—	—	1.330 (0.673–2.629)	0.413	—	—	1.388 (0.672–2.865)	0.672
Employment status	—	—	0.670 (0.332–1.355)	0.265	—	—	0.677 (0.325–1.409)	0.297
Level of formal education (ref: elementary/middle/high school)								
Bachelor’s degree	—	—	1.027 (0.544–1.940)	0.934	—	—	1.122 (0.570–2.206)	0.740
Postgraduate education	—	—	1.557 (0.658–3.781)	0.307	—	—	1.578 (0.620–4.062)	0.336
Perceived family income	—	—	0.611 (0.347–1.079)	0.090	—	—	0.549 (0.301–1.003)	0.051
Urban green areas (ref: Q1)								
Q2	—	—	—	—	0.440 (0.214–0.904)	0.025	0.517 (0.241–1.111)	0.091
Q3	—	—	—	—	0.868 (0.416–1.813)	0.707	1.074 (0.495–2.333)	0.856
Q4	—	—	—	—	0.366 (0.153–0.869)	0.024	0.712 (0.271–1.871)	0.491
Forests (ref: Q1)								
Q2	—	—	—	—	1.311 (0.636–2.704)	0.463	1.198 (0.556–2.579)	0.645
Q3	—	—	—	—	1.611 (0.773–3.355)	0.203	1.382 (0.630–3.031)	0.419
Q4	—	—	—	—	1.002 (0.482–2.084)	0.995	1.234 (0.564–2.702)	0.599
Water (ref: Q1)								
Q2	—	—	—	—	0.517 (0.255–1.050)	0.068	0.499 (0.236–1.054)	0.068
Q3	—	—	—	—	1.296 (0.636–2.642)	0.476	1.276 (0.591–2.755)	0.535
Q4	—	—	—	—	0.984 (0.466–2.077)	0.966	0.939 (0.425–2.076)	0.877
Continuous urban fabric (ref: Q1)								
Q2	—	—	—	—	0.725 (0.351–1.509)	0.393	0.732 (0.339–1.584)	0.428
Q3	—	—	—	—	0.861 (0.408–1.817)	0.695	0.987 (0.447–2.178)	0.974
Q4	—	—	—	—	0.942 (0.362–2.450)	0.903	2.836 (0.843–9.555)	0.092
Nagelkerke R^2^	0.022		0.140		0.103		0.213	
−2 Log likelihood	383.1		355.9		364.3		338.3	

## Data Availability

The data presented in this study are available from the corresponding author upon request. The data are not publicly available due to patient confidentiality.

## Supplementary Materials

The following supporting information can be downloaded at: https://www.mdpi.com/article/10.3390/healthcare13121475/s1, S1: Pregnant Women’s Air Quality Perception, Impact, and Adaptation Questionnaire—P-AIR-Q.
