# Supplementary material for: From Perception to Action: Air Pollution Awareness and Behavioral Adjustments in Pregnant Women in Serbia
Source: Healthcare (Basel). 2025 Jun 19;13(12):1475. doi: 10.3390/healthcare13121475 (PMC12193646; doi:10.3390/healthcare13121475)
Supplement: Supplementary file 1 [file healthcare-13-01475-s001.zip › healthcare-3673354-supplementary.pdf]

Supplementary Material.

S1: Pregnant Women's Air Quality Perception, Impact, and Adaptation Questionnaire - P-AIR-Q form

P-AIR-Q

**Participant No. ___________**		
**S1**	How old are you?	___________________________________
**S2**	Do you smoke?	☐ No<br>☐ Yes<br>☐ Former smoker
**S3**	Does anyone in your household smoke?	☐ No<br>☐ Yes<br>☐ Yes, but not in my vicinity/room
**S4**	What is your current residential address?	_________________________________<br>_________________________________<br>_________________________________
**S5**	What type of residence do you live in?	☐ House<br>☐ Apartment building
**S6**	Which floor do you live on?	☐ Ground to 2nd<br>☐ 3rd to 5th<br>☐ 6th and above
**S7**	How are windows in your home oriented?	☐ Overlooking the street<br>☐ Overlooking the yard<br>☐ Both
**S8**	What is your employment status?	☐ Employed<br>☐ Unemployed
**S9**	What was the last degree of education you obtained?	☐ Elementary/middle school<br>☐ High school<br>☐ Bachelor's degree<br>☐ Postgraduate degree
**S10**	How would you describe your family's income?	☐ Good<br>☐ Fair<br>☐ Poor
**S11**	What is your general opinion regarding air quality in your home city or neighborhood?	☐ Good<br>☐ Fair<br>☐ Poor
**S12**	Do you check the air quality reports provided by official sources?	☐ No<br>☐ Yes
**S13**	Do you consult any unofficial sources reporting on air quality?	☐ No<br>☐ Yes<br>Which ones?<br>_____________________
**S14**	Do you change your habits when the air quality is low?	☐ No<br>☐ Yes, I avoid opening windows<br>☐ Yes, I avoid leaving home<br>☐ Yes, I use air purifiers at home<br>☐ Yes, I wear a face mask outdoors<br>☐ Yes, other<br>How?<br>_____________________

**S15**	Were you informed about recommendations regarding air pollution? If yes, what was the source of these recommendations?	☐ No<br>☐ Yes, I was informed by my doctor<br>☐ Yes, I was informed through media/internet<br>☐ Yes, I was informed by other sources<br>Which ones? ________________________
**S16**	Do you have any symptoms that you would attribute to air pollution?	☐ No<br>☐ Yes
**S17**	If yes, how would you describe these symptoms?	______________________________________
**S18**	Are these symptoms more common during a certain season?	☐ Yes, in spring<br>☐ Yes, in summer<br>☐ Yes, in autumn<br>☐ Yes, in winter<br>☐ No, my symptoms appear all year round
**S19**	Do these symptoms subside when you're indoors?	☐ No<br>☐ Partially<br>☐ Yes
**S20**	Does air pollution cause you any fear or psychological discomfort?	☐ No<br>☐ Yes
